# Identification and Molecular Characterization of YsaL (Ye3555): A Novel Negative Regulator of YsaN ATPase in Type Three Secretion System of Enteropathogenic Bacteria *Yersinia enterocolitica*

**DOI:** 10.1371/journal.pone.0075028

**Published:** 2013-10-04

**Authors:** Rakesh Chatterjee, Pranab Kumar Halder, Saumen Datta

**Affiliations:** Structural Biology and Bioinformatics Division, Council of Scientific and Industrial Research-Indian Institute of Chemical Biology, Kolkata, West Bengal, India; Centre National de la Recherche Scientifique, Aix-Marseille Université, France

## Abstract

Type Three Secretion (T3S) ATPases are involved in delivery of virulent factors from bacteria to their hosts (through injectisome) in an energy (ATP) dependent manner during pathogenesis. The activities of these ATPases are tightly controlled by their specific regulators. In *Yersinia enterocolitica*, YsaN was predicted as a putative ATPase of the Ysa-Ysp Type Three Secretion System (T3SS) based on sequence similarity with other T3S ATPases. However detailed study and characterization of YsaN and its regulation remains largely obscure. Here, in this study, we have successfully cloned, over-expressed, purified and characterized the molecular properties of YsaN from *Yersinia enterocolitica.* YsaN acts as a Mg^2+^ dependent ATPase and exists in solution as higher order oligomer (dodecamer). The ATPase activity of oligomeric YsaN is several fold higher than the monomeric form. Furthermore, by employing *in silico* studies we have identified the existence of a negative regulator of YsaN- a hypothetical protein YE3555 (termed ‘YsaL’). To verify the functionality of YsaL, we have evaluated the biochemical and biophysical properties of YsaL. Purified YsaL is dimeric in solution and strongly associates with YsaN to form a stable heterotrimeric YsaL-YsaN complex (stoichiometry- 2∶1). The N terminal 6–20 residues of YsaN are invariably required for stable YsaL-YsaN complex formation. YsaL inhibited the ATPase activity of YsaN with a maximum inhibition at the molar ratio 2∶1 (YsaL: YsaN). In short, our studies provide an insight into the presence of YsaN ATPase in *Yersinia enterocolitica* and its regulator YsaL. Our studies also correlate the functionality of one of the existing protein interaction networks that possibly is indispensable for the energy dependent process of Ysa-Ysp T3SS in pathogenic *Yersinia enterocolitica.*

## Introduction

Type Three Secretion Systems (T3SSs) are commonly used by many gram negative bacteria for the delivery of toxic bacterial proteins into eukaryotic cells [1], [2,] [3], [44]. Functional T3SS comprises of following groups of proteins *viz* – chaperones, translocators, effectors, apparatus proteins and regulators - each endowed with special functions. The major structural component of T3SS assembly is a hollow needle like structure, commonly known as the ‘needle complex’ or ‘injectisome [3], [44]. This injectisome spans the bacterial inner and outer membrane, protruding outside the cell for delivery of toxins during infection upon contact with host cells [4]. Such a needle structure was first observed in *Salmonella* [5] and *Shigella* [6] using Cryo-Electron Microscopy. Apart from these organisms, similar structures have been also visualized in *Yersinia enterocolitica* [7], *Escherichia coli* [8] and *Pseudomonas aeruginosa* [9]. The apparatus proteins are essential for the assembly of these needle complexes and display similarities among different bacterial species [10], [11]. In contrast to the apparatus proteins and chaperones, the translocators, effectors and the regulators are relatively less conserved in different microorganisms employing T3SS [12], [13]. The chaperones protect the translocators and the effectors in the bacterial cytosol and guide them to the injectisome for secretion [12]–[15]. These translocators and effectors are then channeled through the conduit of the needle complex into their respective hosts in an energy dependent manner by T3S ATPases [16]–[18].The proton motive force coupled with ATP hydrolysis facilitates such translocation [16], [19], [20]. Evolved from the β subunit of F_0_F_1_ ATP synthases, T3S ATPases share significant sequence homology with other closely related species and flagellar system ATPases with a characteristic feature of Walker A and Walker B motifs [19]. These ATPase are important for virulence and are tightly regulated in the cytosol for proper functionality of T3SS [18], [21], [33]–[36].

*Yersinia enterocolitica* is a psychrophilic zoonotic pathogen, which causes acute gastritis leading to symptoms like bloody diarrhea, fever and abdominal pain in young children - commonly known as ‘Yersiniosis’ [22]. Besides these symptoms, arthritis, glomerulonephritis, uvetis and myocarditis also result from chronic *Y. enterocolitica* infections [23]. Of different strains of *Y. enterocolitica*, biovar 1B is highly pathogenic to humans and causes 50% mortality in patients with systemic infection [24]. Infection begins with the ingestion of contaminated food and water. Once inside the gut, the bacteria travel through the M cells into the Payer’s patch of the intestine and translocate anti- phagocytotic proteins by contact dependent T3SS to interfere with the signaling components of the host innate immune response [25]–[27]. To attain full virulence, *Y. enterocolitica* biovar 1B requires two independent T3SSs [28], [29] - a pYV plasmid encoded Ysc-Yop T3SS responsible for the systemic phase of infection and a 200 kb genomic encoded Ysa-Ysp T3SS that causes the gastrointestinal phase of infection. Genes encoding the Ysa-Ysp T3SS are mostly located within the plasticity zone, a genomic region responsible for virulence of *Y. enterocolitica* biovar 1B [30]–[32]. Virulence in Ysc-Yop T3SS is attained by translocation of Yop (Yersinia outer proteins) effectors through injectisome, coupled with YscN mediated ATP hydrolysis [33]. Localized in the peripheral inner membrane, YscN interacts with a number of proteins of the T3SS. Of several interacting partners, YscL interacts with YscN and inhibits ATPase activity of YscN [33]. Apart from *Y. enterocolitica*, such regulations have been extensively studied in the flagellar system of *Salmonella sp* (FliH negatively regulating FliI ATPase activity) [34], T3SS of *Chlamydia pneumonae* (CdsL negatively regulating CdsN ATPase activity) [35], *Escherechia coli* (EscL negatively regulating EscN ATPase activity) [36] and *Xanthomonas campestris* (HrcL negatively regulating HrcN ATPase) [18]. Although the exact mechanism still remains unclear, probably such regulators inhibit ATPase activity by arresting oligomerization. Similar to YscN, genomic encoded YsaN is predicted to be a putative energizer of Ysa-Ysp T3SS in *Y. enterocolitica* biovar 1B [37]. However, to date nothing is known about the regulation of YsaN in *Y. enterocolitica.*

In this report, we have cloned, over-expressed and enzymatically characterized YsaN as a Mg^2+^ dependent oligomeric co-operative ATPase. Further, we have also identified a hypothetical gene YE3555 (we termed ‘YsaL’) using computational analysis and verified its functionality as a negative regulator of YsaN ATPase activity. The role of terminal residues of YsaN in YsaL-YsaN interaction was also investigated.

## Materials and Methods

### Computational Analysis

YsaN (YE3544) was predicted and annotated as a putative T3S ATPase in Yersinia enterocolitica [37]. For identification of the ATPase regulator, YscL (Uniprot ID: A1JUA3), a known Type Three Secretion ATPase regulator, was used as a query to BLAST (www.ncbi.nlm.nih.gov) against the non-redundant database of *Yersinia enterocolitica* O:8. Individual hits were further analyzed in the Pfam database (http://pfam.sanger.ac.uk/). Multiple sequence analysis was performed using ClustalW program and edited in Jalview 2.7. A phylogenetic tree was constructed with average distance based on percentage identity.

### Preparation of Expression Plasmids

*ysaN* (ye3544) full length [amino acids; aa-1–430], *ysaNΔ_ (1–5)_* [aa:6–430; N-terminal 5 residues deleted], *ysaNΔ_ (1–20)_* [aa:21–430; N-terminal 20 residues deleted], *ysaNΔ_ (426–430)_* [aa:1–425; C-terminal 5 residues deleted], *ysaNΔ_ (411–430)_* [aa:1–410; C-terminal 20 residues deleted], *ysaN_(20–410)_* [aa:20–410; 20 each of both N-terminal and C-terminal residues deleted] and *ye3555* (termed as ‘*ysaL*’) were amplified using specific primers (enlisted in **Table 1**) from *Yersinia enterocolitica* ATCC51871 genomic DNA. All amplified products were then cloned in NdeI and XhoI sites of pET28a (+) or pACYC Duet1 (MCS- II) using standard DNA manipulation techniques. Appropiate constructs were transformed into *E. coli* Top 10 (TA host) and selected against specific antibiotics.

**Table 1:** Primers used in the work.

Gene	Sense primer 5′ → 3′	Antisense primer 5′ → 3′
*ysaN*	TTA**CATATG**AATCTCTTTGATAGCT	TTA**CTCGAG**TCAGTTTGCCAGCTCACGGA
*ysaN Δ_(1–5)_*	TTA**CATATG**AGCTGTGCACACCCGT	TTA**CTCGAG**TCAGTTTGCCAGCTCACGGA
*ysaN Δ_(1–20)_*	TTA**CATATG**CCCGCTGCATGGGGTC	TTA**CTCGAG**TCAGTTTGCCAGCTCACGGA
*ysaN Δ_(426–430)_*	TTA**CATATG**AATCTCTTTGATAGCT	TTA**CTCGAG**TCAACGGAGCTGAGCGTGGT
*ysaN Δ_(421–430)_*	TTA**CATATG**AATCTCTTTGATAGCT	TTA**CTCGAG**TCATGCTTGCTGAAAAAGCC
*ysaN_(21–420)_*	TTA**CATATG**CCCGCTGCATGGGGTC	TTA**CTCGAG**TCATGCTTGCTGAAAAAGCC
*ye3555/ysaL*	TTA**CATATG**ATTAAACATCAAGTCA	TTA**CTCGAG**TCACTGATCGCTGCCATTTT

Letters in bold indicate restriction sites for NdeI (sense primer) and XhoI (Antisense primer).

### Protein Production and Purification

Chemically competent *E. coli* BL21 (DE3) (expression host) was transformed individually with appropriate constructs of pET28a (+)-*ysaN* along with all deletion mutants [pET28a (+)-*ysaN Δ_ (1–5)_,* pET28a (+)-*ysaN Δ_ (1–20)_,* pET28a (+)-*ysaN Δ _(411–430)_,* pET28a (+)-*ysaN Δ_ (426–430)_,* pET28a (+)- *ysaN_ (21–410)_*], pET28a (+)-*ysaL* and pACYC Duet-1-*ysaL*. When expressed, all pET28a (+) constructs consisted of an N terminal his tag while constructs cloned in pACYC Duet−1 (MCS- II) were without any tag (details in **Table 2**). To generate YsaL-YsaN complex, pET28a (+)-*ysaN* was co-transformed with pACYC Duet-*ysaL* in chemically competent *E. coli* BL21 (DE3) cells. Transformants were selected in LB (Luria Bertini) agar plates, supplemented with appropriate antibiotics. Overnight cultures were inoculated into 500 ml LB Broth supplemented with specific antibiotics and incubated at 37°C until the OD_600_ reached 0.4–0.6. Recombinant protein productions were initiated by addition of 0.75–0.8 mM IPTG and cultures were further incubated at 25°C for 14–16 hrs. Cells were harvested by centrifugation at 6000× g for 10 min and pellets were stored at −80°C for future use.

**Table 2:** Strains, plasmid and constructs used in the work.

Strains	Features	Source
E.coli Top10- Cloning Host	F^−^ mcrΔ(mrr mutant hsoRMS mutant mcrBC) Φ80lacZΔM15 ΔlacX74 deoRrecA1 araD139∶(araA leu)7697 galU galK rpsL endA1 nupG	Invitrogen
E.coli BL21(DE3) – Expression host	F^−^ ompT hsds_b_ (r_b_^−^m_b_^−^)gal dcm (DE3)	Novagen
*Yersinia enterocolitica* ATCC*51871	Clinical isolates sharing >98.5% similarity with *Yersinia enterocolitica* 8081, serotype O: 8, biovar 1B.	ATCC*
**Plasmids and Constructs**	**Features**	**Source**
pET28a (+)	Expression plasmid with N-terminal His_6_ tag	Novagen
pACYC Duet-1	Expression plasmid with N-terminal His_6_ tagin MCS-I and C-terminal S-tag in MCS-II	Novagen
pET28a (+)-*ysaN*	Full length ysaN in pET28a(+)	This work
pET28a (+)-*ysaNΔ_(1–5)_*	Deletion construct of pET28a (+)-ysaN (residues 6–430)	This work
pET28a (+)-*ysaNΔ_(1–20_*_)_	Deletion construct of pET28a (+)-ysaN (residues 21–430)	This work
pET8a (+)- *ysaNΔ_(426–430_*_)_	Deletion construct of pET28a (+)-ysaN (residues 1–425)	This work
pET28a (+)-*ysaNΔ_(411–430)_*	Deletion construct of pET28a (+)-ysaN (residues 1–410)	This work
pET28a (+)-*ysaN_(21–410)_*	Deletion construct of pET28a (+)-ysaN (residues 21–410)	This work
pET28a (+)-*ye3555/ysaL*	Full length ye3555/ysaL in pET28a (+)	This work
pACYC Duet-1 *ye3555/ysaL*	Full length ye3555/ysaL in pACYC Duet-1 (MCS-II)	This work

* ATCC-American Type Culture Collection.

#### Purification of YsaN-His and untagged-YsaL-YsaN-His complex

Cells containing YsaN-His were thawed on ice and resuspended in sonication buffer (25 mM Tris pH 8.0, 300 mM NaCl, 2% glycerol, 5 mM Imidazole pH 8.0). 2 mM PMSF (Phenyl methyl sulfonyl fluoride) was added to the suspension prior to sonication. The resuspended pellet was then lysed with a 30 sec pulse/30 sec interval cycle for 5–6 times, using a Q-Sonica 125 sonicator. The sonicated samples were centrifuged at 12000× g for 30–45 min to remove insoluble materials. Although a part of overexpressed YsaN-His formed inclusion bodies, a considerable amount of the protein was recovered from the supernatant. For YsaN-His, the supernatant was passed through Ni-NTA resin column pre-equilibrated with the equilibration buffer (25 mM Tris, 300 mM NaCl, 2%glycerol, 10 mM Imidazole). After removing non specific proteins with the wash buffer (25 mM Tris, 300 mM NaCl, 2% glycerol, 37 mM Imidazole), protein was collected using the elution buffer (25 mM Tris, 300 mM NaCl, 2% glycerol, 250 mM Imidazole). Eluates were dialysed against Imidazole removal buffer (25 mM Tris, 300 mM NaCl and 2% glycerol). Similar to YsaN-His, untagged YsaL-YsaN-His complex was purified under reduced salt condition (150 mM NaCl) by Ni-NTA affinity chromatography. The entire purification was performed at 4°C. Protein concentration was estimated by standard Lowry method and samples were subsequently analysed in SDS-PAGE for purity.

#### Purification of YsaL-His, untagged YsaL-YsaN-His complex, YsaN deletion mutants and YsaN-His refolded (YsaN_flr_-His) under denaturing condition

Frozen pellets of YsaL-His, untagged -YsaL, YsaNΔ_ (1–5)_ -His, YsaNΔ_ (1–20)_ -His, YsaN Δ_ (411–430)_ -His, YsaNΔ_ (426–430)_ -His and YsaN_ (21–410)_ -His were thawed on ice and sonicated as described previously. The post sonication pellet was then resuspended in the unfolding buffer (6 M Guanidium-HCl, 25 mM Tris pH 8.0, 1 mM EDTA, 300 mM NaCl) and incubated on ice for 30 min. Equal volume of ice cold refolding buffer (25 mM Tris,300 mM NaCl, 15% glycerol) was added and further diluted ten times. The suspensions were centrifuged at 13000× g for 45 min and the supernatant was dialyzed against Guanidium removal buffer (25 mM Tris, 50 mM NaCl) with three changes to facilitate refolding. All refolded samples were then purified by Ni-NTA affinity chromatography as described earlier and dialysed in Imidazole removal buffer subsequently. Post-sonication supernatant of YsaN-His was also purified under denaturing condition as mentioned earlier and termed YsaN_flr_-His. Untagged YsaL was highly pure after SEC and did not require further downstream purification (data not shown). For interaction study, refolded YsaN deletion mutants (His tagged) and YsaN_flr_-His were incubated with refolded untagged YsaL in 1∶1 ratio and incubated at 4°C for 1 hr. Next the incubated mixtures were purified by Ni-NTA affinity chromatography column (Ni-NTA pull down assay) similar to untagged YsaL- YsaN-His complex. All samples were purified at 4°C and analyzed in SDS PAGE for purity. Protein concentration was estimated by standard Lowry method.

### Chemical Crosslinking Experiments

The stoichiometric composition of recombinant YsaN-His, YsaL- His and untagged YsaL-YsaN- His complex was determined by crosslinking experiments. Experiments were carried out with crosslinkers - Sulfo-EGS [ethylene glycol bis (sulfosuccinimidylsuccinate) or EGSS] (Pierce Biotechnology, Inc.) and gluteraldehyde (Fischer scientific) as per the manufacturer’s instructions. Prior to crosslinking, all the proteins (Ni-NTA Eluates of YsaN-His, YsaL-His and size exclusion chromatography eluates of untagged YsaL-YsaN-His) were dialyzed in sodium phosphate buffer (50 mM sodium phosphate buffer pH 8.0, 150 mM NaCl). The crosslinking reaction using YsaN- His (200 µg) was performed in a total reaction volume of 50 µl. The reaction was carried out in the presence of EGSS (0.5 mM) at 25°C and aliquots were withdrawn after 2 min and 5 min, respectively. Similar to YsaN-His, crosslinking reactions of YsaL-His (100 µg) was performed in a total volume of 30 µl in the presence of 2 µL of 1.5% of freshly prepared glutaraldehyde. Reaction was incubated at 25°C for 5 min. In case of untagged-YsaL-YsaN-His complex, 200 µg of purified complex was subjected to crosslinking reaction in the presence of 1.0 mM EGSS. Aliquots were withdrawn after 5 min and 10 min intervals. For all crosslinking experiments a suitable control (i.e. proteins incubated without crosslinkers) was used. The reaction was terminated by the addition of sample loading buffer for SDS PAGE. Following cross linking, samples were subjected to SDS-PAGE analysis.

### Size Exclusion Chromatography

All Ni-NTA purified proteins (YsaN-His, untaggedYsaL-YsaN-His and YsaL-His) were loaded onto a Superdex 200 Hi-load 16/60 pre-packed size exclusion chromatography (SEC) column pre equilibrated with SEC Buffer (25 mM Tris pH 8.0, 100 mM NaCl, 1 mM EDTA) at the concentration 0.2–1.0 mg/ml and with a flow rate of 1 ml/min. Elution was monitored in real-time using absorbance at 280 nm (A_280_) against retention volume in ÄKTAprime plus and elution profiles for each of the proteins were analyzed in Prime View 5.0. Prior to sample loading the column was calibrated with appropriate gel filtration markers [Thyroglobulin (T) 650 kDa (Elution volume for A_280_ maximum: ev- 47.6 ml), Ferritin (F) 440 kDa (ev- 53.4 ml), Aldolase (A) 158.4 kDa (ev- 65 ml), Ovalbumin (O) 43 kDa (ev- 79 ml), Carbonic anhydrase (C) 29 kDa (ev- 83 ml) and Ribonuclease A (R) 14 kDa (ev- 93 ml)]. The column has a void volume of 45 mL. A standard curve was generated with log of Molecular weight (MW) plotted against Elution volume (ml). Molecular weights of the interested proteins were calculated from this curve.

### Dynamic Light Scattering Experiment (DLS)

YsaN-His was dialyzed in imidazole removal buffer and subjected to DLS in Malvern Zetasizer Nano–ZS® spectrophotometer with a protein concentration of 0.7 mg/ml at 25°C. Imidazole removal buffer was used as a control for the experiment. Molecular mass of the sample was calculated using hydrodynamic diameter.

### ThioflavinT (ThT) Assay

A 40 µM of stock solution of Thioflavin T (ThT) was made using molar extinction coefficient (ε_412_ = 35000 M^−1^ cm^−1^). Prior to the experiment, YsaN-His was dialysed in imidazole removal buffer. Next 5 µM of YsaN-His was mixed with 4 µM of ThT solution and incubated for 1 hr at 25°C. Fluorescence emission intensity was monitored at 482 nm (range 470–500 nm) against the excitation of 440 nm. Results were recorded in Hitachi F4500 fluorescence F-7000 FL spectrophotometer. Fluorescence spectra for individual 4 µM of ThT and 5 µM of YsaN-His in imidazole removal buffer were also recorded for comparative analysis. The slit size for all experiment was 10 nm and each of the experiments was repeated for 5 times.

### ATPase Activity Assay Using Pi-Malachite Green Method

The ATPase activity of YsaN-His was measured in terms of released inorganic phosphate (P_i_) by the malachite green method [38] with minor modifications. The reaction was carried out in 50 mM Tris pH- 8.0 and 10 mM MgCl_2_ (Sigma) in a total reaction volume of 5 ml. Fixed concentration of YsaN-His (2 µM) was incubated with variable concentration of ATP in order to maintain different YsaN-His - ATP ratios (1∶100, 1∶150, 1∶200, 1∶250, 1∶275, 1∶300, 1∶350, 1∶500, 1∶600, 1∶700, 1∶800, 1∶1000, 1∶1200, 1∶1500, 1∶1750 and 1∶2000). For each reaction, 700 µl of reaction mixture was withdrawn at different time points and added to 200 µl of malachite green reagent. After 2 min incubation, 100 µL of 15% citric acid (Merck Germany) was added to each reaction mixture and incubated for another 25 min. Samples were diluted to 1∶10 ratio and inorganic phosphate (P_i_) OD was measured at 630 nm. The concentration of free inorganic phosphate (P_i_) was calculated from standard curve. All measurements of released P_i_ were done at 30°C and substrate corrections were made wherever necessary. Specificity for divalent cations (10 mM MgCl_2_, 10 mM MnCl_2_, 10 mM CaCl_2_, 10 mM ZnCl_2_,), substrates [(500 µM dATP- deoxyadenosine triphosphate, 500 µM dGTP- deoxyguanosine triphosphate, 500 µM dCTP- deoxycytidine triphosphate and 500 µM dTTP- deoxythymididne triphosphate)], pH (100 mM Sodium acetate: pH 5.0–5.5, 100 mM MES: pH 6.0–6.5, 100 mM HEPES: pH 7.0–7.5, 100 mM Tris : pH 8.0–8.5 and100 mM BICINE: pH 9.0–9.5) and temperature (16–51°C with 7°C interval) was also determined for YsaN-His. For YsaN_flr_-His and all YsaN deletion constructs, 2 µM of enzymes were incubated with 500 µM of ATP and P_i_ released was measured as described previously. In case of regulation studies, YsaL-His was added in increasing concentration to enzyme reaction mixtures (YsaN-His +500 µM ATP in 50 mM Tris pH 8.0 and 10 mM MgCl_2_) as such the molar ratios of YsaL-His and YsaN-His were 0∶1, 0.5∶1, 1∶1, 1.5∶1, 2∶1, 2.5∶1 and 3∶1. 0.5 µM of YsaN-His dodecamer and YsaN-His monomer was individually incubated with 350 µM of ATP. Release of inorganic phosphate (P_i_) was quantified similarly.

### Secondary Structure Determination by Circular Dichroism

For circular dichroism (CD) experiments YsaL-His, YsaN-His, YsaN_flr_-His and different YsaN deletion mutants were dialyzed against phosphate buffer (50 mM sodium phosphate pH 8.0 100 mM NaCl). Far UV CD spectra for each of the samples were recorded from 190 nm to 250 nm in Jasco J-815 spectrophotometer at 1 nm interval, using 0.1 cm pathlength cuvette with a protein concentration of 20 µg/ml. For each CD spectrum, ellipticity in machine units [θ] was plotted against wavelength (nm). Secondary structure within the protein was calculated by using the algorithms available in DichroWeb server.

### Binding kinetics Analysis by Surface Plasmon Resonance

The binding of YsaL with YsaN, YsaN_flr_ and all the his tagged deletion mutants were determined by Surface Plasmon Resonance (SPR) in Biacore−3000 (Biacore® - GE Healthcare). YsaN-His, YsaN_flr_-His and all his tagged-deletion mutants of YsaN (ligands) were immobilized on NTA-chip (Biacore® - GE Healthcare) saturated with Ni^2+^ solution as recommended by the manufacturer. Kinetics and binding analysis were performed in SPR running buffer (10 mM HEPES pH 7.4, 150 mM NaCl, 50 µM EDTA) and ligands were injected at a flow rate 5 µl/min for 10 minutes. Analyte (untagged- YsaL) was injected in the concentrations of 5 nM, 10 nM, 20 nM and 50 nM and sensogram was monitored for another 70 mins. All experiments were performed at 25°C. Sensogram analysis was done in Biaeval software v 4.1.

## Results

### YsaN is an Oligomeric Magnesium-dependent walker-type ATPase

**Figure 1:**
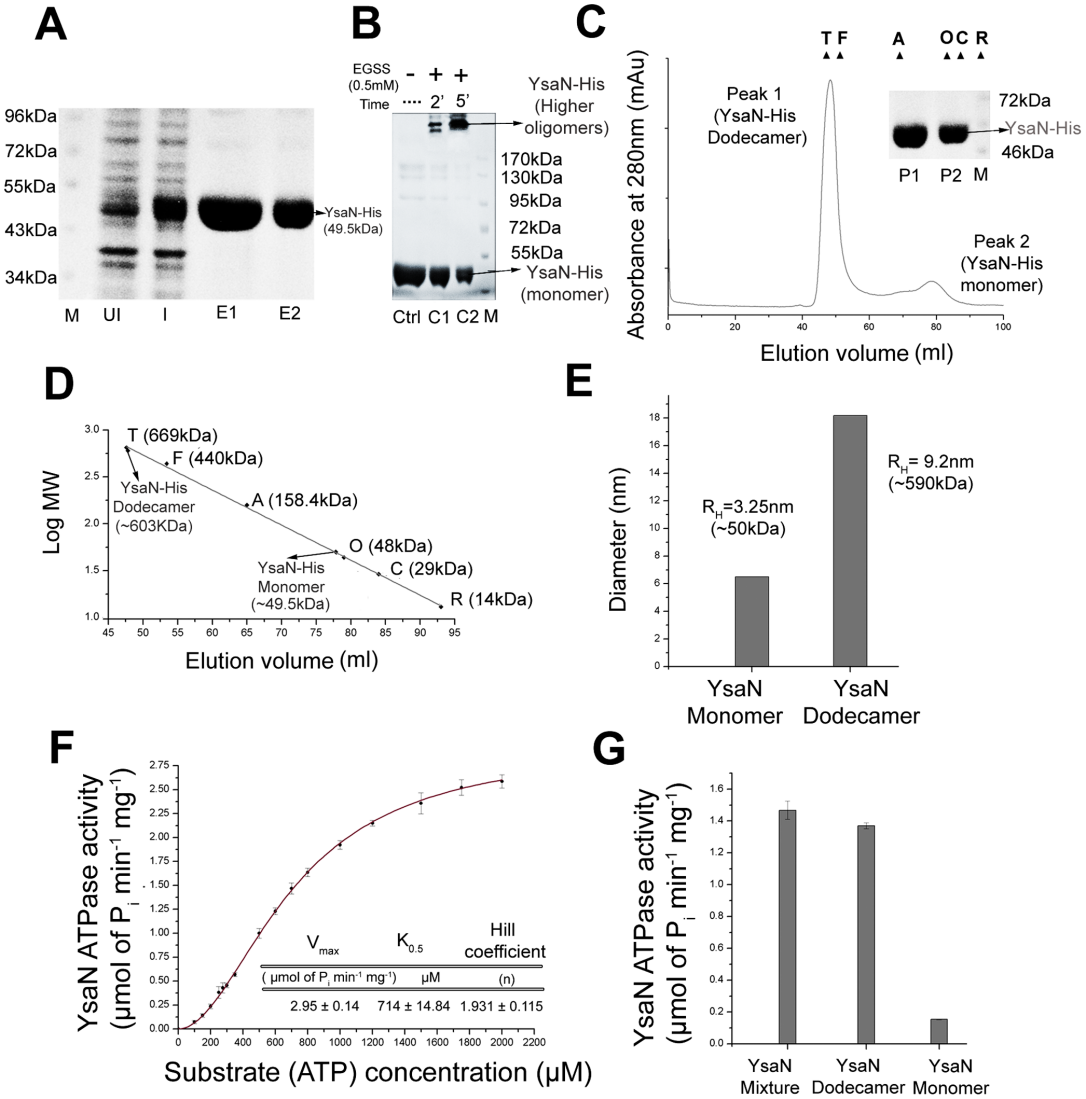
Oligomerization analysis and enzymatic characterization of YsaN ATPase. (**A**) SDS PAGE analysis of YsaN-His after purification by Ni-NTA affinity chromatography: M- Marker, UI- Uninduced, I- induced, E1 and E2- Ni-NTA eluates. (**B**) Chemical crosslinking profile of YsaN-His in SDS-PAGE using Sulfo - EGS (EGSS) - Ctrl-Control, C1 and C2- crosslinking of YsaN-His using 0.5 mM EGSS incubated for 2 min and 5 min respectively, M-Marker. (**C**) Size exclusion chromatography (SEC) profile of YsaN-His in pre-calibrated Superdex 200 hi-load16/60 column (GE Healthcare). Molecular weight standards are indicated with triangles: Thyroglobulin (T) –669 kDa, Ferritin (F) –440 kDa, Aldolase (A) –158.4 kDa, Ovalbumin (O) –48 kDa, Carbonic anhydrase (C) –29 kDa and Ribonuclease A (R) –14 kDa. SDS PAGE analysis of Gel filtration profile: P1 (Peak1) - YsaN-His Dodecamer and P2 (Peak2) - YsaN-His monomer, M - marker **(inset)**. (**D**) Molecular mass estimation from size exclusion profile of YsaN-His using the above known standards. Peak1 from SEC profile corresponds to ∼603 kDa (Dodecamer) and peak 2 corresponds to ∼49.5 kDa (Monomer). (**E**) Measurement of hydrodynamic diameter of YsaN-His using Dynamic Light Scattering. Hydrodynamic radii (R_H_) of YsaN corresponded to monomer and dodecamer. (**F**) ATPase activity of Ni-NTA eluates of YsaN-His measured in terms of Phosphate (Pi) released per minute per mg of protein. Hill fit equation provided the kinetic parameters - V_max_, K_0.5_ and hill coefficient (n). (**G**) Relative ATPase activity of YsaN Dodecamer and YsaN Monomer separated after SEC. YsaN mixture (monomer and oligomer) was used as a control.

YsaN was predicted as a putative ATPase and found to be responsible for translocation of secretory proteins like YspC, YspD, YspA and YspB [37]. However, the biochemical and biophysical properties of YsaN remains largely unexplored. In this context, YsaN from *Y.enterocolitica* was cloned, overexpressed and characterized. Recombinant YsaN was purified to homogeneity as a his tag fusion with molecular mass of ∼49.5 kDa (**Figure 1A** **)**. In a sulfo-EGS (EGSS) crosslinking experiment, YsaN-His was detected only in a higher oligomeric state **(** **Figure 1B** **)**. To analyze this higher oligomeric state and distinguish it from aggregative artifacts, YsaN-His was subjected to SEC in a Superdex 16/60 Hi-Load column followed by SDS-PAGE analysis. Results indicated that YsaN-His existed in both as a dodecamer (∼603 kDa) and in a monomeric state (∼49.5 kDa), with the dodecameric form being the predominant one **[** **Figure 1C, 1C** **(inset) and 1D]**. YsaN-His was also subjected to DLS and the hydrodynamic radius (R_H_) was used to estimate the molecular weight. The peaks had R_H_ corresponding to ∼50 kDa (10% of sample) and ∼590 kDa (90% of sample) corresponding to monomeric and oligomeric forms respectively **(** **Figure 1E** **)**. Thioflavin T is a common fluorophore used to identify β-amyloid formation and is detected by a characteristic red shift of its emission spectra. When YsaN-His was incubated with Thioflavin T (ThT) no binding was observed **(figure S1)** and thereby suggesting the non-aggregative nature. Although, such experiment is helpful for studying and distinguishing aggregative artifacts from oligomers, we cannot completely rule out the possibility of YsaN-His to form aggregates. All the above experiments had good agreement with SEC data and indicated that YsaN existed in dodecameric and monomeric forms. Such higher oligomeric state (dodecameric state) is exhibited by many T3S ATPases for elevated enzymatic activity [**41**].

Similar to other ATP hydrolyzing enzymes, YsaN consists of Walker A (residue 160–167) - ‘AXXXXGKT(S) (X- any aminoacids) and Walker B (residue 246–251) – ‘hhhhDS’ (h- hydrophobic aminoacids) motifs. Enzymatic studies of YsaN indicated that it has the highest preference for deoxy ATP (dATP) as substrate among all deoxynucleotides **(figure S2A)**. Using ATP as a substrate, the role of the divalent ions was also verified. Like its plasmid counterpart YscN and other T3S ATPases, Mg^2+^ was essential for ATP hydrolysis by YsaN-His **(figure S2B)**. Addition of metal ion chelator (EDTA) in ATPase buffer with Mg^2+^ or ATPase buffer without Mg^2+^ resulted in several fold reduction in ATPase activity. Optimum activity was observed in Tris pH 8.0 **(figure S2C)** at 30°C **(figure S2D)**. ATPase activity of YsaN-His was comparable at 30°C and 37°C. Owing to the precipitation nature of YsaN-His at 37°C, all experiments were conducted at 30°C. YsaN-His mediated ATP hydrolysis was reduced considerably below pH 6.5 and above pH 8.5 and at temperatures above 42°C and below 25°C, respectively.

When plotted against ATP (substrate) concentration, ATPase activity of YsaN-His (Ni-NTA purified) followed sigmoidal kinetics and satisfied Hill equation **(** **Figure 1F** **)**. It had a mean K_0.5_ of 714±14.84 µM and a V_max_ of 2.95±0.14 µmol of P_i_ released min^−1^ mg^−1^, data which are comparable to other T3S ATPases – InvC, YscN, EscN and FliI. The Hill coefficient (n) of 1.931±0.115 indicated positive cooperativity.

Ni-NTA purified YsaN-His existed in both dodecameric and monomeric states. To examine their functionality, relative ATPase activity of both these forms was estimated. Therefore, YsaN-His oligomer – monomer mixture (i.e. Ni-NTA eluates) was separated by SEC and individual states were immediately analysed for their relative ATP hydrolysing capacity. YsaN-His dodecamer was more active in hydrolysing ATP as compared to its monomeric form. YsaN-His monomer-oligomer mixture was used as a control in this experiment. **(** **Figure 1G** **)**.

### Identification of YE3555 as a Negative Regulator of YsaN Using Computational Methods

Since all T3S ATPases are controlled by specific negative regulators, the existence of a negative regulator was examined in this Ysa-Ysp T3SS of *Y.enterocolitica*. Protein BLAST analysis using YscL as the query against *Yersinia enterocolitica* O:8 non-redundant database, retrieved 11 hits. Proteins, clustered in the same protein family or clan, are usually endowed with similar structures and functions. So, all of these hits were analyzed for their respective protein families in the Pfam database **(** **Table 3** **)**. Only 2 hits were found to be in the HrpE/YscL/FliH family and belonged to the same clan (CL0255). Since YP_001006743.1 was already annotated as a flagellar assembly protein- FliH, so YP_001007713.1 (hypothetical protein YE3555) was of primary attention. When compared with 27 unique protein sequences from the HrpE/YscL/FliH family using multiple sequence analysis **(figure S3)**, YE3555 was found to be in a cluster with YscL, PscL, SctL and HrpE (all known T3S ATPase negative regulators) in the phylogram. The other cluster consisted of proteins from different bacterial species that largely belonged to flagellar assembly protein, FliH **(figure S4A)**. This suggested that YE3555 had close evolutionary relationship with the T3S ATPase regulators. Primary sequence analysis of YE3555 revealed the presence of the repeat sequences- AXXXGXXXG and AXXXA (X representing any amino acids), a characteristic feature of the YscL/FliH ATPase regulator family proteins [39] **(figure S4B and S4C)**. Furthermore, the presence of ye3555 within the 200 kb Ysa locus of *Y. enterocolitica* and in the close vicinity of ysaN (ye3544), indicated that it might be an integral part of this genomic encoded T3SS in this organism **(figure S4D)**. Such predictions demanded careful and detailed experimental investigation to validate the candidature of YE3555 as a T3S ATPase negative regulator.

**Table 3:** Pfam analysis of BLASTp hits with YscL as a query.

Accession code	Protein family (pfam) number:	Probable protein function
Ref|YP_001006743.1|	PF 02108*	Flagellar assembly protein H
Ref|YP_001006305.1|	PF 02776, PF 00205	Acetolactate synthase catalytic subunit
Ref|YP_001007533.1|	PF 01037, PF 13412	AsnC transcription regulator
Ref|YP_001005995.1|	PF 03461,PF 00271, PF 00270, PF 00259	Transcription repair coupling factor
Ref|YP_001006844.1|	PF 06857	Citrate lyase subunit gamma
Ref|yp_001006481.1|	PF 00126	LysR transcriptional regulator
Ref|YP_001006442.1|	PF 001006	Short chain dehydrogenase
Np 863517.1	PF 00771	Low calcium ion response protein D
Ref|YP_001007713.1|	PF 02108*	Hypothetical protein YE3555
Ref|YP_001004634.1|	PF 03466, PF 00126	LysR transcriptional regulator
Ref|YP_001005611.1|	PF 02264	Hypthetical protein YE1290

* Belonged to same clan HrpE/YscL/FliH and V-type ATPase subunit E (CL 0255).

### YsaL is Dimeric in Solution with Predominant Helical Structure

**Figure 2:**
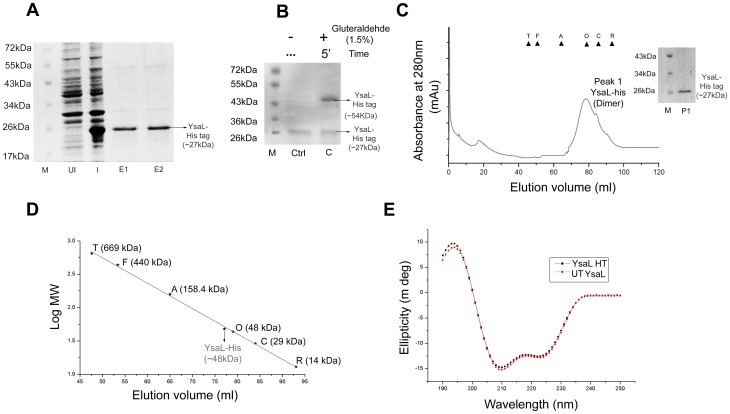
Purification, stoichiometric characterization and secondary structure analysis of YsaL/Ye3555. (**A**) SDS PAGE analysis of refolded YsaL-His after Ni-NTA affinity chromatography: M-Marker, UI- Uninduced, I- induced, E1 and E2- Ni-NTA eluates. (**B**) Chemical crosslinking profile of refolded YsaL-His in SDS-PAGE using 1.5% Gluteraldehyde - M- Marker Ctrl- Control, C- crosslinking of YsaL-His using 1.5% Gluteraldehyde incubated for 5 min. (**C**) Size exclusion chromatography (SEC) profile of refolded YsaL-His in pre-calibrated Superdex 200 hi-load16/60 column (GE Healthcare). Molecular weight standards are indicated with triangles: Thyroglobulin (T) –669 kDa, Ferritin (F) –440 kDa, Aldolase (A) –158.4 kDa, Ovalbumin (O) –48 kDa, Carbonic anhydrase (C) –29 kDa and Ribonuclease A (R) –14 kDa. SDS PAGE analysis of Gel filtration profile: M – marker, P1 (peak1) - YsaL-His **(inset)**. (**D**) Molecular mass estimation from size exclusion profile of refolded YsaL-His using the above known standards. Refolded YsaL-His was dimeric (∼48 kDa). (**E**) Far UV-CD (Circular dichroism) spectra of refolded YsaL-His (HT) and refolded untagged (UT) YsaL.

Based on bioinformatics analysis, which strongly implied YE3555/YsaL to be a negative regulator, we tried to characterize it biochemically and biophysically. Overexpressed YsaL-His was completely insoluble, compelling purification under denaturing condition. Therefore, YsaL-His was refolded and purified to homogeneity by Ni-NTA affinity chromatography. Purity of the sample was analyzed by SDS-PAGE and bands corresponding to ∼27 kDa **(** **Figure 2A** **)** were observed. In a gluteraldehyde-crosslinking experiment, a band with a molecular mass of ∼54 kDa was observed for YsaL-His - probably indicating a homodimeric association **(** **Figure 2B** **)**. This idea was further supported by the observation of a peak in the SEC profile corresponding to ∼48 kDa **[** **Figure 2C, 2C** **(inset) and 2D]**. YsaL-His did not have any ATP hydrolysis capacity as inferred from the malachite green assay (data not shown). Secondary structure analysis revealed that YsaL in His tagged or untagged form had a high helical content (α helix –63–64%) and considerable β-sheets –11–12%. The remaining part was assumed to form random coils or β-turns **(** **Figure 2E** **)**. Experimental analysis by Far UV CD was in close agreement with secondary structure prediction (**Table S1)**.

### YsaL co-purifies with YsaN in Soluble form and Negatively Regulates YsaN ATPase Activity by Reducing Oligomerization

**Figure 3:**
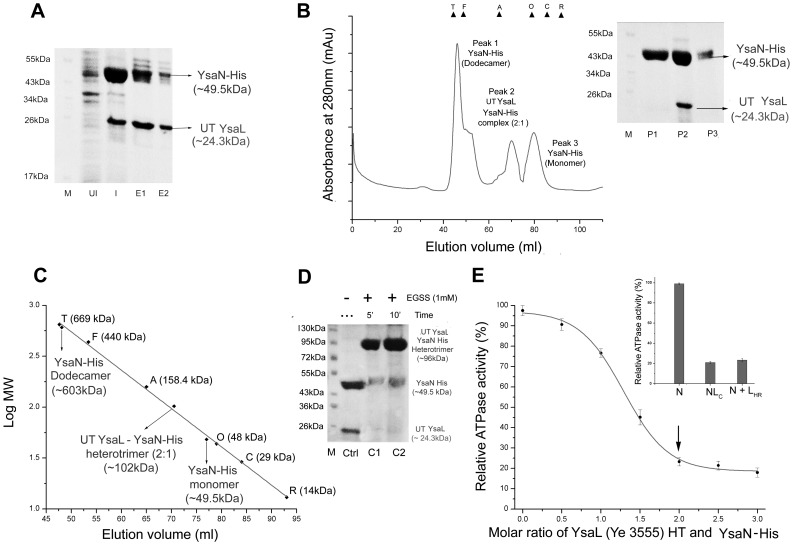
Co-Purification, stoichiometry and functional analysis of YsaL-YsaN complex. (**A**) SDS-PAGE analysis of YsaL-YsaN complex after co-purification in Ni-NTA affinity chromatography: M-Marker, UI- Uninduced, I- induced, E1 and E2- Ni-NTA eluates of untagged YsaL-YsaN-His complex. (**B**) Size exclusion chromatography (SEC) profile of untagged YsaL-YsaN-His complex in pre-calibrated Superdex 200 hi-load16/60 column (GE Healthcare). Molecular weight standards are indicated with triangles: Thyroglobulin (T) –669 kDa, Ferritin (F) –440 kDa, Aldolase (A) –158.4 kDa, Ovalbumin (O) –48 kDa, Carbonic anhydrase (C) –29 kDa and Ribonuclease A (R) –14 kDa. SDS PAGE analysis of Gel filtration profile: M – marker, P1 (Peak1) - YsaN-His dodecamer P2 (Peak2) - untagged (UT) YsaL-YsaN-His heterotrimer and P3 (Peak3) - YsaN-His monomer **(inset)**. (**C**) Molecular mass estimation from size exclusion profile of untagged YsaL-YsaN-His complex using the known molecular weight standards. Peak1 corresponds to YsaN-His (Dodecamer) - ∼603 kDa, Peak2 corresponds to heterotrimeric assembly of untagged YsaL-YsaN-His complex (2∶1) - ∼102 kDa and Peak3 corresponds to YsaN-His (monomer) - ∼49.5 kDa. (**D**) Chemical crosslinking profile of untagged (UT) YsaL-YsaN-His complex in SDS-PAGE using Sulfo - EGS (EGSS) - Ctrl-Control, C1 and C2-crosslinking of untagged YsaL-YsaN-His using 1.0 mM EGSS incubated for 5 min and 10 min respectively, M-Marker. (**E**) Relative ATPase activity (%) with increasing molar ratio of refolded YsaL-His/Ye3555 and YsaN-His. Maximum inhibition of ATPase activity occurs in the ratio 2∶1 shown by arrow. Relative ATPase activity of untagged YsaL-YsaN-His complex (NL_C_) and YsaN-His incubated with YsaL-His refolded (N +L_HR_) in the molar ratio 2∶1. YsaN-His (N) was used as a control **(inset)**.

Bioinformatical studies strongly support YsaL as negative regulator of ATPase activity. However, to establish this experimentally, we co-expressed pET28a-YsaN and pACYC Duet-YsaL in *E.coli* BL21 (DE3) cells to produce YsaN-His and untagged YsaL, respectively. Ni-NTA pull down experiments from the IPTG induced cell lysates confirmed that untagged YsaL (∼24.3 kDa) associates with YsaN-His (∼49.5 kDa) and the complex was co-purified **(** **Figure 3A** **)**. Untagged YsaL (refolded), which did not bind to Ni-NTA was used as a negative control (data not shown) for the experiment. For further purification, eluates from the Ni-NTA affinity chromatography were subjected to SEC followed by SDS PAGE analysis. SEC gave a peak corresponding to a heterotrimeric assembly (untagged YsaL: YsaN-His - 2∶1) for untagged YsaL-YsaN-His complex (∼102 kDa) **[** **Figure 3B and 3C** **]**. Excess YsaN-His - both in dodecameric and monomeric states were also detected by SEC and SDS PAGE analysis **[** **Figure 3B** **(inset)]**. Chemical crosslinking of untagged YsaL-YsaN-His complex with Sulfo - EGS produced band corresponding to ∼96 kDa in SDS PAGE **(** **Figure 3D** **)**, which corroborated the SEC data.

These experimental studies so far, suggested that YsaN and YsaL interact with each other and exists as complex in solution. Additional experiments were also performed to validate whether, YsaL inhibits ATPase activity of YsaN or not. Results from malachite green assay showed YsaL/YE3555 (either his tag or untagged) inhibited 73% of ATPase activity of YsaN-His in the molar ratio 2∶1 (YsaL: YsaN) **[** **Figure 3E** **]**. Further increase in molar ratio of YsaL-YsaN (ratio 2.5∶1 and 3∶1) had little or no effect in reduction of ATPase activity. ATPase activities of soluble untagged YsaL-YsaN-His complex and that of refolded YsaL (His tag) incubated with soluble YsaN-His in the molar ratio 2∶1, were highly comparable **[** **Figure 3E** **(inset)]**. Thus, refolded YsaL-His was equally competent in repressing ATPase activity of YsaN when compared with soluble untagged YsaL-YsaN-His complex. Addition of control protein BSA or thermally denatured untagged YsaL to YsaN-His did not reduce ATPase activity as confirmed from malachite green assay (data not shown).

### Role of Terminal Amino Acids of YsaN in YsaL-YsaN Interactions

**Figure 4:**
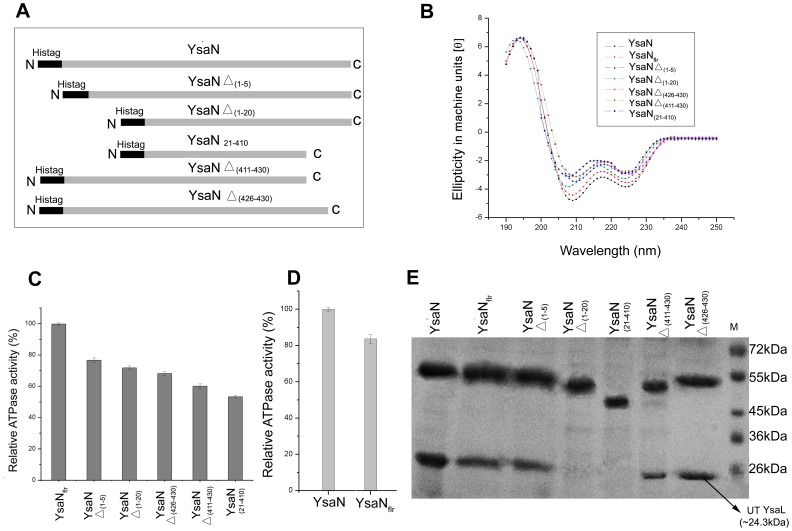
Identification of critical residues of YsaN for stable YsaL-YsaN complex formation. (**A**) Schematic diagram of deletion mutant constructs of YsaN. (**B**) Far-UV CD spectra of his tagged deletion mutants of YsaN [YsaN Δ_(1–5)_, YsaN Δ_(1–20)_, YsaN Δ_(426–430)_, YsaN Δ_(411–430)_ and YsaN_(21–410)_] compared with full length YsaN (YsaN-His) and refolded YsaN full length (YsaN_flr_-His). (**C**) Relative ATPase activity (%) of deletion mutants of YsaN with YsaN_flr_-His as a control. (**D**) Relative ATPase activity (%) of YsaN-His with YsaN_flr_-His. (**E**) SDS-PAGE profile of Ni-NTA pull-down assay of deletion mutants of YsaN (His tagged) with untagged YsaL; YsaN _Δ(1–20)_ and YsaN_ (21–410)_ does not bind to untagged (UT) YsaL. M-denotes molecular weight marker.

Terminal amino acids are reported to be vital for the ATPase- regulator assembly. In FliH-FliI interaction study, a FliH down regulates FliI ATPase activity. In *Salmonella typhimurium*, the N-terminal 20 amino acids of FliI are necessary for successful FliI-FliH interaction [40]. To study the role of terminal amino acids of YsaN in YsaL-YsaN interactions, some deletion mutants were generated **(** **Figure 4A** **)**. However, the bottleneck for this experiment was that all deletion mutants of YsaN localized into inclusion bodies upon expression. So we purified YsaN deletion mutants under denaturing conditions by Ni-NTA his-tag affinity chromatography (details in materials and methods). All the eluates were subjected to Far UV CD **(** **Figure 4B** **)** and were analyzed for their respective ATP hydrolysis capacity **(** **Figure 4C** **)**. In this respect, YsaN-His was refolded from post sonication supernatant (termed YsaN_flr_-His) and was used as a control for comparative analysis of YsaN deletion mutant **(** **Figure 4D** **)**. Deletion mutants only with significant secondary structures **(** **Figure 4B** **)** and considerable ATPase activity (>50% compared to YsaN_flr_) were considered for further analysis **(** **Figure 4C** **)**. Compared to YsaN-His, YsaN_flr_-His had ∼85% activity **(** **Figure 4D** **)** and displayed significantly similar secondary structure in CD experiments **(** **Figure 4B** and **Table S1).** All the C terminal [YsaNΔ _(426–430)_ -His and YsaNΔ _(411–430)_ -His] deletion mutants had reduced ATPase activity but showed comparable secondary structures with respect to the N- terminal deletion mutants [YsaNΔ_ (1–5)_-His and YsaNΔ_ (1–20)_-His] in the far-UV CD experiments **(** **Figure 4B and 4C** **)**. YsaN_ (21–410)_-His had lowest ATPase activity and secondary structural signal in far UV CD.

**Figure 5:**
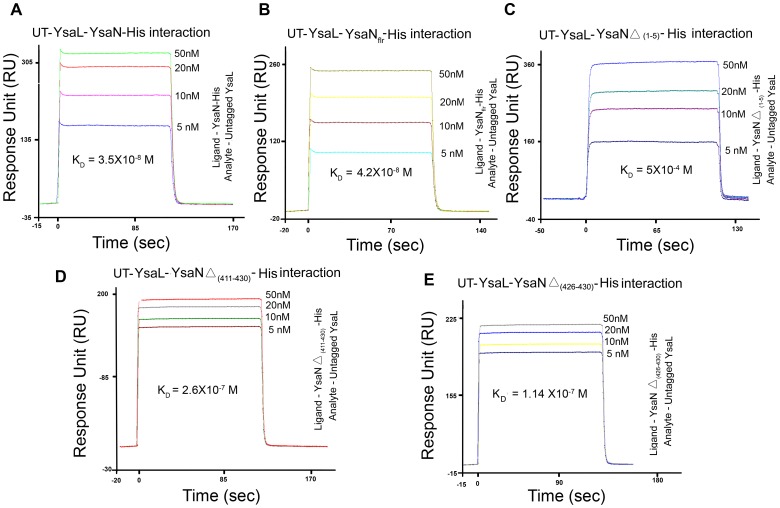
Binding propensity of YsaL with YsaN wild type and different deletion mutants using Surface Plasmon resonance. Interactions of untagged (UT) YsaL with (**A**) YsaN-His (**B**) YsaN_flr_-His (**C**) YsaN Δ_ (1–5)_ -His (**D**) YsaN Δ_ (411–430)_ -His and (**E**) YsaN Δ _(426–430)_-His. Untagged YsaL was used as an analyte in the concentration 5 nM, 10 nM, 20 nM and 50 nM.

Untagged YsaL co-purified with YsaNΔ _(426–430)_-His, YsaNΔ _(411–430)_ -His and YsaNΔ_ (1–5)_ -His. However, YsaNΔ_ (1–20)_ -His and YsaN_(21–410)_ -His failed to interact with untagged YsaL as evident from Ni-NTA-His pull down assay and SDS PAGE analysis **(** **Figure 4E** **)**.To gain further insight into the binding of deletion mutants of YsaN, SPR analysis was done for each of the mutants using untagged YsaL as the binding counterpart **(Table S2)**. The SPR data when fitted and compared to the Hill-Langmuir’s standard equation curve, revealed that YsaN-His interacted with untagged YsaL with a K_D_ (Dissociation constant) of 3.5×10^−8^ M **(** **Figure 5A** **).** YsaN_flr_ interacted with untagged YsaL with K_D_ of 4.2×10^−8^ M **(** **Figure 5B** **)**. YsaN-HisΔ _(1–5)_ showed the weakest interaction (K_D_ of 4×10^−4^M) among all the fragments **(** **Figure 5C** **)** while YsaN-HisΔ _(1–426)_ and YsaN-HisΔ _(1–411)_ had K_D_ values in 10^−7^ M range **(** **Figure 5D and 5E** **)**. No binding was observed for YsaN-HisΔ_ (1–20)_ or YsaN-His_ (21–410)_.

## Discussion

**Figure 6:**
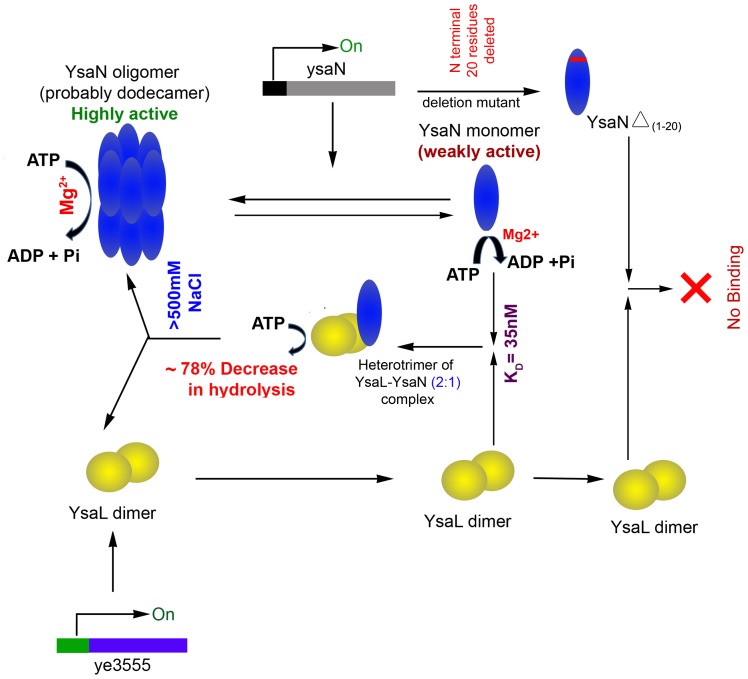
Schematic representation of functionality of YsaN and its regulation by YsaL. YsaN exists in solution as mixture of monomer and higher order oligomer (dodecamer) and act as Mg ^2+^ dependent ATPase. The oligomeric form of YsaN is highly active compared to monomeric form. Computational studies predicted YsaL as a putative ATPase regulator. YsaL exists as dimer in solution and form stable heterotrimeric complex with monomeric YsaN. This results in significantly loss of ATPase activity of YsaN, probably due to loss of oligomeric state. The complex is unstable at high salt concentration (>500 mM NaCl). Furthermore, N-terminal (1–20) residues of YsaN are involved in YsaL-YsaN interaction, as revealed from interaction studies of deletion mutants.

Although the genome-encoded Ysa-Ysp T3SS in *Yersinia enterocolitica* has many genes contributing to its virulence, most of them remain largely uncharacterized and unexplored. Gene knockdown experiments have only envisaged YsaN as a putative ATPase that promotes virulence in this organism by translocating secretary proteins [37]. In this study, we have successfully purified and characterized YsaN as a magnesium-dependent ATP-hydrolyzing enzyme. In fact absence of Mg^2+^ considerably reduced its ATP hydrolysis capacity. Initial studies of YsaN using SEC, DLS and Thioflavin T indicated that YsaN remains predominantly in a dodecameric state with small amount in a monomeric form. Enzymatic analysis further revealed that dodecameric YsaN is more active than its monomer. Such homo-dodecameric assembly accounts for elevated enzymatic activity [41] and is probably essential for translocation of T3SS substrates (effectors/translocators) during infection with *Yersinia enterocolitica* [37]. A common characteristic feature of almost all T3S and flagellar ATPase is that they are tightly controlled within the microbial pathogens to translocate virulence factors when required [18] ^,^ [33]–[36]. In this context existence of such a regulator for YsaN was examined in *Yersinia enterocolitica*. Computational prediction strongly indicated Ye3555/YsaL to be a negative regulator of YsaN. Lack of any consensus sequence made such identification difficult. However, the presence of Glycine- Alanine repeats suggested that this hypothetical protein is probably a member of the YscL/FliH/HrpE family [39]. Refolded YsaL was predominantly a helical protein existing in homodimeric state, which is a common attribute of most T3S and flagellar ATPase regulators [33], [34], [45]. Successful co-purification of untagged-YsaL with YsaN-His by affinity chromatography clearly indicated that YsaL is an interacting partner of YsaN. This interaction was further investigated by SPR, which showed that YsaL associated with YsaN strongly, having a K_D_ of 35 nM. From 1∶1 Hill-Langmuir equation fit in SPR, it was inferred that YsaL interacted with YsaN only in homodimeric state, instead of binding as two separate monomers. SEC analysis and crosslinking experiments indicated that YsaL associates with YsaN in a heterotrimeric form with stoichiometry of 2∶1 (YsaL: YsaN). Enzymatic analysis by malachite green assay revealed that maximum inhibition of YsaN activity by YsaL occurs in the same molar ratio of 2∶1 (YsaL: YsaN). Although the exact mechanism of such regulation remains unclear, it is anticipated that YsaL binds with YsaN and prevents dodecamerization, resulting in inhibition of ATPase activity. Thus YsaN is retained in a weakly activated state (monomer) in the cytosol of *Yersinia enterocolitica* by dimeric YsaL. As compared to N terminal residues, C terminal deletions of YsaN hardly affected stable YsaL-YsaN complex formation but resulted in reduced ATPase activity, probably due to the loss of helical content. N terminal constructs had lower β sheet content and higher ATP hydrolysis capacity, compared to their C terminal counterparts. Interaction studies of YsaN Δ_ (1–5)_ with YsaL using SPR, indicate that such residues might not be directly involved in YsaL-YsaN complex formation rather they play an auxiliary role in this process. From molecular mapping of YsaN using deletion constructs followed by SPR analysis, it is evident that absences of N terminal 6–20 aminoacids (SCAHPSRIHGCLLEA) affect stable YsaL-YsaN complex formation. We reckon that dimeric YsaL interacts with YsaN monomer at the N terminus, inhibiting oligomerization resulting in down regulation of its ATPase activity **(** **Figure 6** **)**.

This is our first attempt at successfully identifying and characterizing YsaL (YE3555) as a negative a regulator of YsaN, as evident from the above experiments. The presence of such regulator and ATPase also indicate independent functionality of this relatively new Ysa-Ysp T3SS. However, to reach any conclusions, experimental verification is necessary. Future studies with detailed insight into the exact mechanism by YsaL-YsaN complex formation during gastrointestinal phase of infection of *Yersinia enterocolitica* will be essential in understanding the pathogenicity of this organism. In the Ysc-Yop T3SS, YscL probably tethers YscN to the C ring of the injectisome with the help of YscQ, one of the structural components of the C ring antechamber [33], [42]. YscK might act as a cargo delivery protein and probably allows localization of translocator/effector-chaperone complexes at the base of injectisome prior to their delivery into the host [43]. Sequence analysis strongly indicated YsaQ and YsaK in the Ysa-Ysp T3SS are close homologues of YscQ and YscK respectively. Such proteins might play an important role in YsaN mediated virulence, although it requires further experimental verification. Future investigations revealing the existence of such protein network in *Yersinia enterocolitica* will help in proper explanation of the delivery of virulent factors through this relatively unexplored Ysa-Ysp T3SS.

## Supporting Information

Figure S1 **ThioflavinT (ThT) assay of YsaN-His.** Binding of Thioflavin T with YsaN-His was measured with respect to fluorescence intensity against wavelength. Fluorescence intensity of Buffer with ThT (blue), YsaN-His+ Buffer (Black) and YsaN-His +ThT +buffer (red) are depicted.
(TIF)

Figure S2 **Physiological parameters affecting YsaN ATPase activity. (A)** Substrate specificity of deoxy nucleotides, **(B)** Cations effect, **(C)** pH and **(D)** Temperature.
(TIF)

Figure S3 **Multiple Sequence analysis of Ye3555/ysaL with 27unique sequences from YscL/HrpE/FliH family.** Sequences belonging to flagellar system are marked in green and T3SS negative regulators are marked in light yellow. Both of them are boxed in two groups. The name of the protein is depicted within short identifiers with their respective organisms- Ah- *Aeromonas hydrophilla*, Vc- *Vibrio cholereae*, Xc- *Xanthomonas campestris*, Yp-Yersinia pestis, Yp*-Yersinia pestis strain Pestoides, Ypy yersinia psudotuberculosis, Ye-*Yersinia enterocolitica*,Ye_0∶3 *Yersinia enterocolitica 0∶3*,*Ye_0;8 Yersinia enterocolitica 0∶8* Pmi-*Proteus mirabilis*, St- *Salmonella typhimurium*, Ymo- *Yersinia mollaretii*, Pr- *Providencia rettegeri*, Pl/Phlu- *Photorhabdus luminiscence*, Pru- *Providencia rustigianii*, Sg- *Sodalis glossinidius* (strain morsitans), Clp- *Chlamydia pneumonae*, Ps- *Pseudomonas syringae*, Pa- *Pseudomonas aeruginosa (PA14)*,Pal- *Pseudomonas alcaligens* Pf-*Pseudomonas fluorescens*, Sf- *Shigella flexneri*, Bp-*Burkholderia pseudomallei*, *Lph* - *Legionella pneumophila*, Ec57- *Escherechia coli* 0∶57. Predicted secondary structure of YsaL along with logo showing incidence of amino acid at a particular position.
(TIF)

Figure S4 **Identification and prediction of ye3555 as a negative regulator of Ysa-Ysp T3SS using computational analysis. (A)** Phylogram of YE 3555 in comparison to 27 unique sequences from YscL/HrpE/FliH protein family (ATPase negative regulators). Cluster coloured in green corresponds to ATPase negative regulator of flagellar system while T3S ATPase negative regulators are coloured in light yellow. The name of the protein is indicated with their organisms in abbreviated forms- Ah- *Aeromonas hydrophilla*, Vc- *Vibrio cholereae*, Xc- *Xanthomonas campestris*, Yp-*Yersinia pestis,* Yp*-Yersinia pestis strain Pestoides, Ypy yersinia psudotuberculosis, Ye-*Yersinia enterocolitica*,Ye_0∶3 *Yersinia enterocolitica 0∶3*,*Ye_0;8 Yersinia enterocolitica 0∶8* Pmi-*Proteus mirabilis*, St- *Salmonella typhimurium*, Ymo- *Yersinia mollaretii*, Pr- *Providencia rettegeri*, Pl/Phlu- *Photorhabdus luminiscence*, Pru- *Providencia rustigianii*, Sg- *Sodalis glossinidius* (strain morsitans), Clp- *Chlamydia pneumonae*, Ps- *Pseudomonas syringae*, Pa- *Pseudomonas aeruginosa (PA14)*,Pal- *Pseudomonas alcaligens* Pf-*Pseudomonas fluorescens*, Sf- *Shigella flexneri*, Bp-*Burkholderia pseudomallei*, *Lph* - *Legionella pneumophila*, Ec57- *Escherechia coli* 0∶57. FliH of Lph shared greater similarity with T3S ATPase negative regulators. Values within the tree denote distances from nearest node. **(B)** Primary sequence analysis of YE3555 with 27 sequences showing the region of AXXX (GXXXG)_n_ XXXA repeat (n denotes number of GXXXG repeats). Ala (A) is marked in red and Gly (G) in green and _n_ denotes number of repeats. **(C)** AXXXA ‘repeat type’ sequence (A marked in orange) of YE3555 in comparison to its 27 orthologues. **(D)** Gene location of ye3555 and ysaN in the Ysa-ysp locus. Arrowheads indicate the direction of transcription (Adapted from NCBI Genome- NC_008800). Ye3555 is marked in red triangle in all the figures.
(TIF)

Table S1 **Comparative analysis of secondary structure of YsaN and YsaL using Circular Dichroism and bioinformatical prediction.**
(DOCX)

Table S2 **Binding kinetic parameters of untagged YsaL (analyte) to his tagged YsaN and its deletion mutants.** YsaN and its deletion mutants were immobilized as ligands and YsaL was used as an analyte with varying concentrations of 5 nM, 10 nM, 20 nM and 50 nM.
(DOCX)
