# Supplementary figures and images for: Identification and Molecular Characterization of YsaL (Ye3555): A Novel Negative Regulator of YsaN ATPase in Type Three Secretion System of Enteropathogenic Bacteria Yersinia enterocolitica
Source: PLoS One. 2013 Oct 4;8(10):e75028. doi: 10.1371/journal.pone.0075028 (PMC3790809; doi:10.1371/journal.pone.0075028)

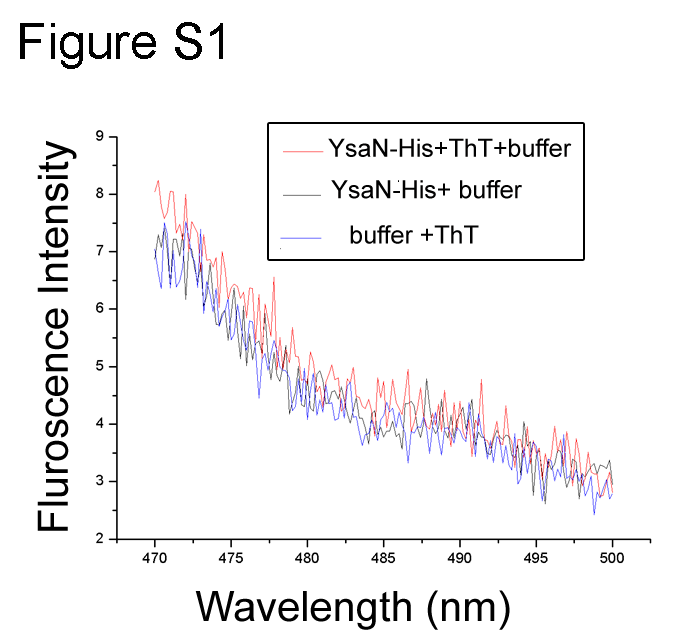

Supplement: Figure S1 — ThioflavinT (ThT) assay of YsaN-His. Binding of Thioflavin T with YsaN-His was measured with respect to fluorescence intensity against wavelength. Fluorescence intensity of Buffer with ThT (blue), YsaN-His+ Buffer (Black) and YsaN-His +ThT +buffer (red) are depicted. (TIF) [file pone.0075028.s001.tif]

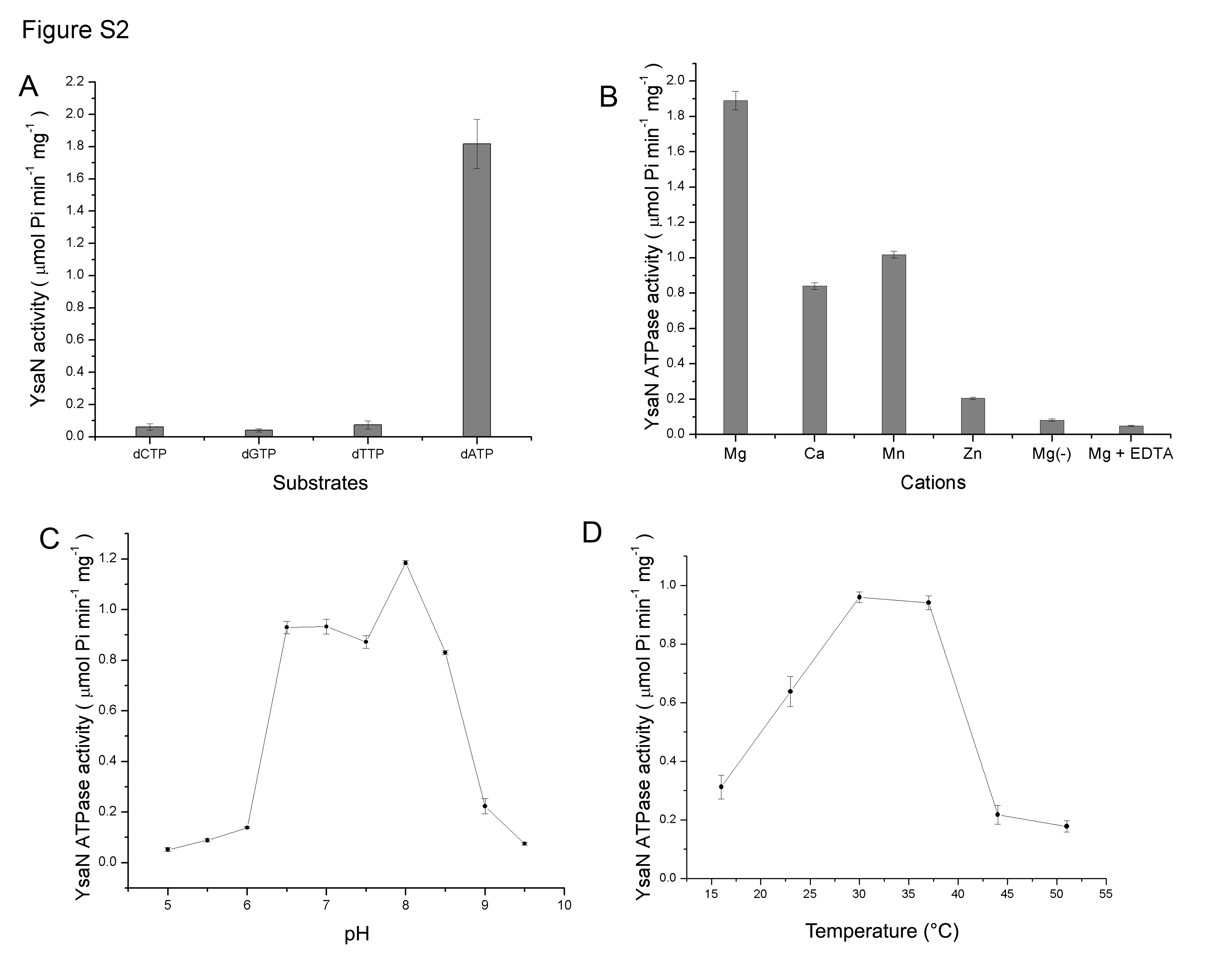

Supplement: Figure S2 — Physiological parameters affecting YsaN ATPase activity. (A) Substrate specificity of deoxy nucleotides, (B) Cations effect, (C) pH and (D) Temperature. (TIF) [file pone.0075028.s002.tif]

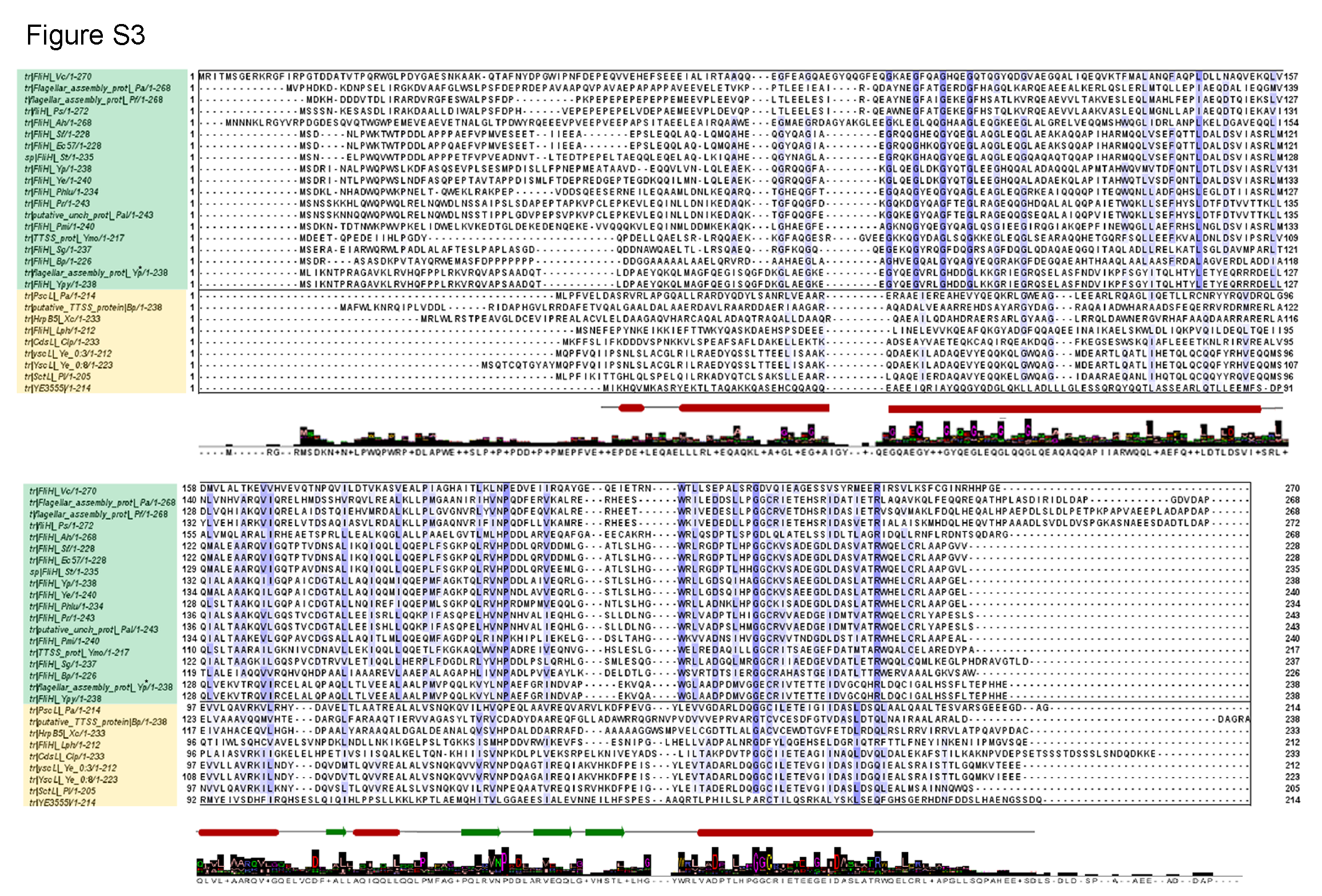

Supplement: Figure S3 — Multiple Sequence analysis of Ye3555/ysaL with 27unique sequences from YscL/HrpE/FliH family. Sequences belonging to flagellar system are marked in green and T3SS negative regulators are marked in light yellow. Both of them are boxed in two groups. The name of the protein is depicted within short identifiers with their respective organisms- Ah- Aeromonas hydrophilla, Vc- Vibrio cholereae, Xc- Xanthomonas campestris, Yp-Yersinia pestis, Yp*-Yersinia pestis strain Pestoides, Ypy yersinia psudotuberculosis, Ye-Yersinia enterocolitica,Ye_0∶3 Yersinia enterocolitica 0∶3,Ye_0;8 Yersinia enterocolitica 0∶8 Pmi-Proteus mirabilis, St- Salmonella typhimurium, Ymo- Yersinia mollaretii, Pr- Providencia rettegeri, Pl/Phlu- Photorhabdus luminiscence, Pru- Providencia rustigianii, Sg- Sodalis glossinidius (strain morsitans), Clp- Chlamydia pneumonae, Ps- Pseudomonas syringae, Pa- Pseudomonas aeruginosa (PA14),Pal- Pseudomonas alcaligens Pf-Pseudomonas fluorescens, Sf- Shigella flexneri, Bp-Burkholderia pseudomallei, Lph - Legionella pneumophila, Ec57- Escherechia coli 0∶57. Predicted secondary structure of YsaL along with logo showing incidence of amino acid at a particular position. (TIF) [file pone.0075028.s003.tif]

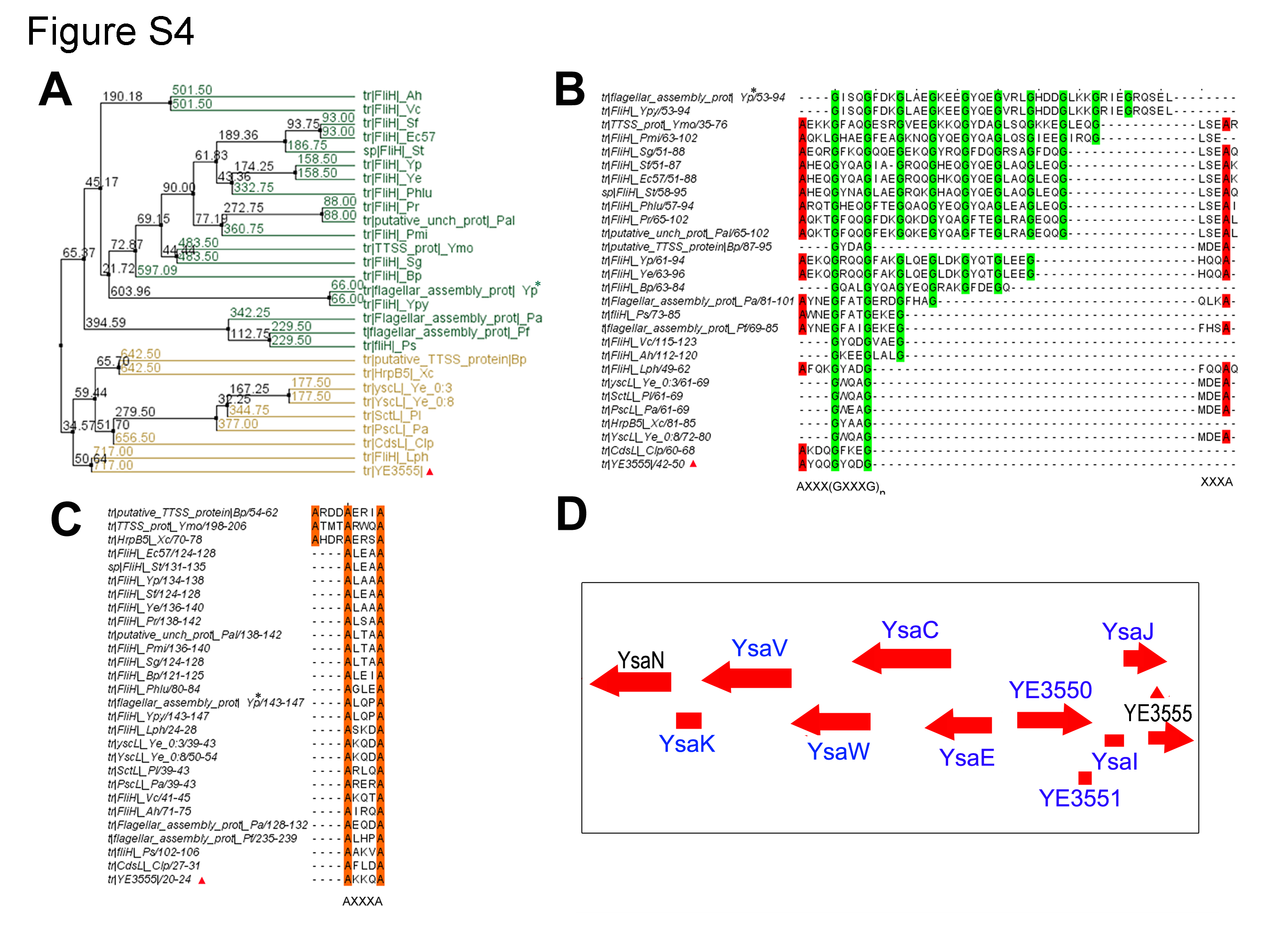

Supplement: Figure S4 — Identification and prediction of ye3555 as a negative regulator of Ysa-Ysp T3SS using computational analysis. (A) Phylogram of YE 3555 in comparison to 27 unique sequences from YscL/HrpE/FliH protein family (ATPase negative regulators). Cluster coloured in green corresponds to ATPase negative regulator of flagellar system while T3S ATPase negative regulators are coloured in light yellow. The name of the protein is indicated with their organisms in abbreviated forms- Ah- Aeromonas hydrophilla, Vc- Vibrio cholereae, Xc- Xanthomonas campestris, Yp-Yersinia pestis, Yp*-Yersinia pestis strain Pestoides, Ypy yersinia psudotuberculosis, Ye-Yersinia enterocolitica,Ye_0∶3 Yersinia enterocolitica 0∶3,Ye_0;8 Yersinia enterocolitica 0∶8 Pmi-Proteus mirabilis, St- Salmonella typhimurium, Ymo- Yersinia mollaretii, Pr- Providencia rettegeri, Pl/Phlu- Photorhabdus luminiscence, Pru- Providencia rustigianii, Sg- Sodalis glossinidius (strain morsitans), Clp- Chlamydia pneumonae, Ps- Pseudomonas syringae, Pa- Pseudomonas aeruginosa (PA14),Pal- Pseudomonas alcaligens Pf-Pseudomonas fluorescens, Sf- Shigella flexneri, Bp-Burkholderia pseudomallei, Lph - Legionella pneumophila, Ec57- Escherechia coli 0∶57. FliH of Lph shared greater similarity with T3S ATPase negative regulators. Values within the tree denote distances from nearest node. (B) Primary sequence analysis of YE3555 with 27 sequences showing the region of AXXX (GXXXG)n XXXA repeat (n denotes number of GXXXG repeats). Ala (A) is marked in red and Gly (G) in green and n denotes number of repeats. (C) AXXXA ‘repeat type’ sequence (A marked in orange) of YE3555 in comparison to its 27 orthologues. (D) Gene location of ye3555 and ysaN in the Ysa-ysp locus. Arrowheads indicate the direction of transcription (Adapted from NCBI Genome- NC_008800). Ye3555 is marked in red triangle in all the figures. (TIF) [file pone.0075028.s004.tif]
