# Supplementary material for: Identification and Molecular Characterization of YsaL (Ye3555): A Novel Negative Regulator of YsaN ATPase in Type Three Secretion System of Enteropathogenic Bacteria Yersinia enterocolitica
Source: PLoS One. 2013 Oct 4;8(10):e75028. doi: 10.1371/journal.pone.0075028 (PMC3790809; doi:10.1371/journal.pone.0075028)
Supplement: Table S1 — Comparative analysis of secondary structure of YsaN and YsaL using Circular Dichroism and bioinformatical prediction. (DOCX) [file pone.0075028.s005.docx]

Table S1. Comparative analysis of secondary structure of YsaN and YsaL using Circular Dichroism and bioinformatical prediction

| Proteins | Secondary structure from CD analysis (%) | | |
| --- | --- | --- | --- |
|  | Helix (h) | Sheet (s) | Coils/ turns (c) |
| YsaN | 58 | 20 | 22 |
| YsaN_flr_ | 56 | 19 | 25 |
| YsaN Δ _(1-5)_ | 55 | 15 | 30 |
| YsaN Δ _(1-20)_ | 54 | 11 | 35 |
| YsaN _(21-410)_ | 51 | 10 | 29 |
| YsaN Δ _(411-430)_ | 47 | 18 | 35 |
| YsaN Δ _(425-430)_ | 49 | 16 | 34 |
| Untagged YsaL | 64 | 10 | 26 |
| YsaL His-tag | 63 | 10 | 27 |

| Protein | Secondary structure prediction (in %) | | | | | | | | |
| --- | --- | --- | --- | --- | --- | --- | --- | --- | --- |
|  | Psipred | | | Jpred | | | Dsc | | |
|  | Helix | Strand | Coil | Helix | Strand | Coil | Helix | Strand | Coil |
| YsaN | 60 | 21 | 19 | 59 | 20 | 21 | 61 | 21 | 18 |
| YsaL | 68 | 12 | 20 | 67 | 14 | 19 | 68 | 14 | 18 |
