# Supplementary material for: Identification and Molecular Characterization of YsaL (Ye3555): A Novel Negative Regulator of YsaN ATPase in Type Three Secretion System of Enteropathogenic Bacteria Yersinia enterocolitica
Source: PLoS One. 2013 Oct 4;8(10):e75028. doi: 10.1371/journal.pone.0075028 (PMC3790809; doi:10.1371/journal.pone.0075028)
Supplement: Table S2 — Binding kinetic parameters of untagged YsaL (analyte) to his tagged YsaN and its deletion mutants. YsaN and its deletion mutants were immobilized as ligands and YsaL was used as an analyte with varying concentrations of 5 nM, 10 nM, 20 nM and 50 nM. (DOCX) [file pone.0075028.s006.docx]

Table S2. Binding kinetic parameters of untagged YsaL (analyte) to his tagged YsaN and its deletion mutants. YsaN and its deletion mutants were immobilized as ligands and YsaL was used as an analyte with varying concentrations of 5nM, 10nM, 20nM and 50nM.

| Ligand | | Analyte | Forward rate -k_a_ (M^-1^s^-1^) | Backward rate -k_d_ (s^-1^) | Association constant K_A_ (M^-1^) | Dissociation constant K_D_ (M) |
| --- | --- | --- | --- | --- | --- | --- |
| YsaN | Untagged YsaL | | 3.45 X 10^3^ | 1.19 X 10^-4^ | 2.9 X 10^7^ | 3.5 X 10^-8^ |
| YsaN_flr_ | Untagged YsaL | | 4.54 X 10^3^ | 1.91X 10^-5^ | 2.38 X 10^8^ | 4.2 X 10^-8^ |
| YsaN Δ _(1-5)_ | Untagged YsaL | | 2.19 X 10^2^ | 1.08 X 10^-2^ | 2.03 X 10^4^ | 5 X 10^-4^ |
| YsaN Δ _(1-20)_ | Untagged YsaL | | NBD | NBD | NBD | NBD |
| YsaN _(21-410)_ | Untagged YsaL | | NBD | NBD | NBD | NBD |
| YsaN Δ _(411-430)_ | Untagged YsaL | | 5.1 X 10^2^ | 1.32 X 10^-4^ | 3.86 X 10^6^ | 2.6 X 10^-7^ |
| YsaN Δ _(426-430)_ | Untagged YsaL | | 2.01 X 10^2^ | 2.28 X 10^-4^ | 8.82 X 10^5^ | 1.14 X 10^-7^ |

NBD- No Binding
